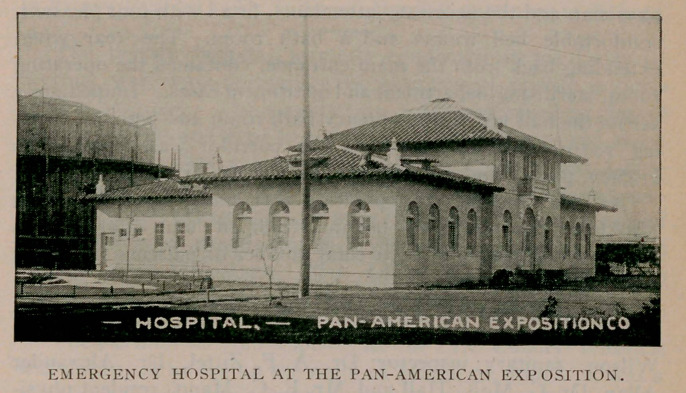# A Century of Medical History in the County of Erie.—1800–19001Note.—The last chapter of this history was published in the Buffalo Medical Journal, April, 1899, which brought the record down to that time. These supplementary chapters are now presented, that the history may be carried from that date forward to the end of the XIXth Century, thus completing it.—W. W. P.

**Published:** 1902-02

**Authors:** William Warren Potter

**Affiliations:** Buffalo, N. Y.


### A Century of Medical History in the County of Erie.—1800–1900.[1]

By WILLIAM WARREN POTTER, M. D., Buffalo, N. Y.

*Pioneer Physicians—Medical Societies—Medical Colleges—Hospitals—Medical Journals—Women Physicians—History of Homeopathy—Medical Officers of the Civil War—Individual Members of the Profession.*

### SUPPLEMENT.

### VIII.—ALUMNI ASSOCIATION MEETINGS.

SOMETIME during the winter of 1899 the officers in charge of the Alumni Association of the Medical Department of the University of Buffalo conceived the idea of holding a midwinter reunion. This conception was carried to a most successful conclusion on Wednesday evening, February 22, 1899.

The selection of this patriotic day for the gathering of the sons and daughters of the college proved most appropriate, and a

1. NOTE.—The last chapter of this history was published in the BUFFALO MEDICAL JOURNAL, April, 1899, which brought the record down to that time. These supplementary chapters are now presented, that the history may be carried from that date forward to the end of the XIXth Century, thus completing it.—W. W. P.

large gathering of physicians and friends of the college met and were agreeably entertained on the occasion referred to.

The executive committee, Dr A. T. Lytle, chairman, and Drs. G. W. Wende, H. U. Williams, Charles Cary, Eli H. Long and A. T. Kerr, associates, conducted an admirable program. The music was good, the supper served in the library was excellent, and the literary part of the entertainment proved most satisfactory.

The hour appointed was 8.30 p.m., and soon afterward the corridors and rooms were well filled with an enthusiastic throng of men and women.

After an exchange of greetings, the guests were conducted through the various laboratories where interesting details were observed, each teacher in charge being at his post to point out or explain some of the newer methods of instruction in vogue. The State Laboratory, with Dr. H. R. Gaylord in immediate charge, where investigations relating to the origin and propagation of cancer were going on, proved of special interest to many. This had been established the previous year under the supervision of Dr. Roswell Park, an appropriation by the legislature having been made for the purpose.

The chemic, microscopic, anatomic, physiologic and pathologic laboratories were thrown open, and it was the opinion of the visitors that nothing more complete than these could be found in any medical institution in this country.

In Alumni Hall, Prof. H. M. Hill exhibited stereopticon views of some of the work that was under study and investigation in these laboratories. These ceremonies occupied attention up to the hour of 9.30 o'clock, which had been appointed for the beginning of the literary exercises.

Dr. M. D. Mann, dean of the medical faculty, was presented and delivered an address in which he set forth the aims and hopes of the faculty, in the course of which he made a strong plea for the endowment of the chairs of anatomy, physiology, chemistry, pathology and bacteriology. He also extended a cordial welcome to the medical alumni of Niagara University to the benefits and pleasures to membership in the association.

Dr. Z. J. Lusk, '75, of Warsaw, N.Y., president of the alumni association, was introduced by the chairman and responded in a short and fitting address. Dr. Mann then introduced Dr. W. G. Taylor, of Buffalo, the last president of the alumni association of Niagara University, who made an appropriate speech.

The address of the evening was to have been delivered by Dr. Frederick Peterson, of New York, but owing to illness he was obliged to recall his promise, which he did in a most appropriate and witty letter of regret, that Dr. Lytle read in full. In Dr. Peterson's stead Dr. William Warren Potter, of Buffalo, addressed the assembled audience for about twenty minutes, after which adjournment was had to the library, where an hour was spent at supper, during which the orchestra played many strains of excellent music. This successful reception was commented upon by all present and a desire expressed for the permanent establishment of the mid-winter reunion.

The second annual reception was held at Alumni Hall, Thursday evening, February 22, 1900. A large audience, generously besprinkled with ladies, graced the occasion and the hall was appropriately decorated with patriotic emblems, reminding all that Washington's birthday had been appropriated by the faculty as permanent University day.

The dean of the faculty, Dr. M. D. Mann, was greeted with a round of applause when he took the chair and introduced the speaker of the evening, Dr. Frederick Peterson, '79, of New York, whose subject was Practical Psychology. To say that it was interestingly, instructively, even ably dealt with only echoes the universal opinion of every one who heard the distinguished visitor.

The next feature of the evening was an attempt by Madame Bergman, of Buffalo, to demonstrate what is termed psychometry—whatever that may mean. At all events the famous "mind-reader" made sorry work of her attempt to measure the mentalities of several of her prominent auditors. It was easy to see how, by her jugglery with words and by making a few common-place guesses, more or less accurate, the superficially trained—even the superrefined—might be impressed with the wonderful "gifts" of such a person.

Dr. Jason Parker, of Jamestown, who was billed to give a practical demonstration of hypnotism, was more successful. From a group of messenger boys hastily collected, he succeeded in apparently hypnotising a number of them. As an exhibition it was entertaining and succeeded in stimulating speculative thoughts in the minds of many present as to its applicability or utility. Dr. Parker received the applause of his audience many times during his entertaining demonstration.

Then came Prof. Gee Hart, of Buffalo, who delighted everybody by a sleight-of-hand performance, during which he filled the air with half dollars, all of which rained into his own coffers. Frequent applause testified to the interest in this part of the entertainment, which closed the exercises of the evening in Alumni Hall, and then luncheon was served in the library where a pleasant hour was passed.

The regular annual meeting—the 24th—of the Alumni Association held April 25, 1899, was presided over by Dr. Z. J. Lusk, '75, of Warsaw, N.Y.

A paper was read by Dr. William G. Bergtold, of Denver, Col., entitled Human hemoglobin in high altitudes, which was discussed by Drs. Charles G. Stockton, Albert E. Woehnert and A. L. Benedict.

Dr. John H. Pryor read a paper entitled, The relative death-rates of cancer and of consumption, which was discussed by Drs. H. R. Hopkins, DeLancey Rochester, H. R. Gaylord and George E. Blackham.

Dr. George Henry Fox, of the College of Physicians and Surgeons, New York, then presented about 100 stereopticon slides, from his original collection, showing the many phases of cutaneous syphilis. At the same time Dr. Fox indicated in a concise manner their diagnostic features, and to those listening and looking it was equal if not superior to a clinic. The reception of this lecture was enthusiastic and it was discussed by Drs. Charles Cary and Ernest Wende.

At the meeting the classes which had reunions were those of '47, '48, '49, '59, '69, '74, '79, '89, and each class took luncheon by itself at the several Buffalo hotels.

A marked feature of the morning session was an informal reception given by a committee of women physicians to women graduates and the wives of visiting alumni, which was held in the library of the University from 10 until 12.30 o'clock. Afterward luncheon was served and at which several felicitous little speeches were made. The library was appropriately decorated with rugs, palms, flowering plants, flags and the college colors. The reception committee consisted of Ida C. Bender, chairman; Maud J. Frye, Mary I. Denton, Edith M. Stewart, Margaret S. Halleck, Lillian C. Randall, Jeannette P. Himmelsbach, Amelia E. Trant, Marie S. Ross, Alice Ross Bennett, Bina P. Vandenberg, Mary M. Huntley, Cora B. Lattin, Jane W. Carroll, Carro J. Cummings, Alice L. Mitchell and Jane N. Frear.

The annual dinner was served in the banquet room of Music

Hall, which was elaborately decorated with national colors. Alumni from 1847 to 1899 inclusive sat down to a delightful spread; music and college songs filled in the time until the toasts were announced by Edward N. Brush, as toastmatter, in his usual entertaining manner. The list was as follows: Forty-niners, '47-8-9, Walter D. O. K. Strong; Twice Two Score, '59, William Warren Potter; Endowment, Eugene A. Smith; Three Decades, '69, Frank A. Jones; A Quarter Century, '74, Robert P. Bush; A Score, '79, William L. Dickinson; Specialism, George H. Fox; A Decade, '89, Robert S. Carr; Today, '99, Frank P. Bingham; Tomorrow, Mary Innes Denton; Farewell, A Poem, Thomas M. Heard.

The twenty-fifth regular session of the Alumni Association was called to order by the President, Dr. William Warren Potter, '59, in Alumni Hall, at 2.30 o'clock p.m., April 27, 1900.

The president delivered his annual address, the subject being Metrorrhagia and the Menopause.

It was discussed by Drs. H. E. Hayd, M. D. Mann and C. C. Frederick.

Dr. Maud J. Frye next read a paper on The symptomatology of early ectopic gestation. It was discussed by Drs. Frederick, Mann, Charles A. L. Reed, of Cincinnati, and Hayd.

Dr. Grover W. Wende followed with an interesting talk on certain skin diseases, illustrated by a large number of most excellent stereopticon views.

Dr. L. A. Weigel, of Rochester, closed the proceedings with an informal talk in the Diagnostic value of the *x*-ray, illustrated by the stereopticon.

In the morning the arriving alumni were greeted by the several committees. After registration and payment of dues came reunions of classes, and informal visiting. They then were taken in charge by the reception committee, Dr. Nelson W. Wilson. chairman of the general committee, and Dr. Margaret S. Halleck, chairman of the women's committee, and escorted to the several rooms set apart for these meetings.

The classes of 1850, 1860, 1870, 1880 and 1890 had appointed meetings for this year. They talked over the past and then each class took luncheon together.

Officers of the class of 1870 were elected as follows: president, Dr. C. B. Kibler, Corry, Pa.; secretary and treasurer, Dr. L. W. Byam, Mumford, N.Y.

Officers of the class of 1880 were elected as follows: president, Dr. Ten Eyck O. Burleson, Bath, N. Y.; secretary,

Dr. C. H. Woodard, Buffalo; treasurer, Dr. C. M. Daniels, Buffalo.

Officers of the class of 1890 were elected as follows: president, Dr. D. J. Tillotson, Rochester; vice-president, Dr. L. J. McAdam, Buffalo; secretary and treasurer, Dr. B. C. Johnson, Buffalo.

The classes of 1850 and 1860 elected no officers.

In the evening the annual dinner was served at the Iroquois. The president, Dr. William Warren Potter, who had held the same office in 1877, presided at the banquet and acted as toastmaster. He inaugurated the speech-making, with a few remarks, closing with a toast to the health of Dr. Roswell Park, who was just then recovering from a grievous illness.

The response was prompt, the audience rose en masse and amidst rousing cheers, flying napkins, and rattling glasses, the health of Dr. Park was eargerly drunk.

The toastmaster, paying little heed to the printed rotation, then announced the sentiments and called upon the speakers in the following order of sequence:

Alma Mater . . . . . . . . . . . . . . . . . . . . . . Dr. Charles G. Stockton.

" He thought as a sage, though he felt as a man."

The Class of '00 . . . . . . . . . . . . . . . . . Dr. William Fred. Powers.

" Think naught a trifle, though it small appear."

The Physician Idealised . . . . . . . . . . . . . . Dr. Cora Billings Lattin.

" It were a journey like the path of heaven,
To help you find them."

Preaching and Practising . . . . . . . . . . . . . . Rev. George B. Richards.

The Pan-American . . . . . . . . Hon. William I. Buchanan, Director-General.

" Lest men suspect your tale untrue,
Keep probability in view."

The Ohio Idea . . . . . . . . . . . . Dr. Charles A. L. Reed, Cincinnati, Ohio.

" There is occasions and causes why and wherefore
in all things."

The election of officers for the ensuing year resulted as follows:

President, Dr. Devillo W. Harrington, Buffalo, class of 1871.

First vice-president, Dr. Frank H. Moyer, Moscow, class of 1872.

Second vice-president, Dr. A. W. Bayliss, Buffalo, class of 1889.

Third vice-president, Dr. C. S. Payne, Liberty, class of 1880.

Fourth vice-president, Dr. Mary J. Slaight, Rochester, class of 1880.

Fifth vice-president, Dr. Clarence King, Machias, class of 1885.

Secretary, Dr. T. H. McKee, Buffalo, class of 1898.

Treasurer, Dr. Herbert U. Williams, Buffalo, class of 1889.

Trustee for five years, Dr. William Warren Potter, Buffalo, class of 1859.

Executive committee, Dr. Albert T. Lytle, Buffalo, class of 1893, chairman; Dr. Grover W. Wende, Buffalo, class of 1889, and Dr. Abram T. Kerr, Buffalo, class of 1897.

### IX.—THE HOSPITALS.

### Buffalo Hospital of the Sisters of Charity.

Sister M. Florence, who for twenty-five years had charge of the Sisters' of Charity Hospital, at Buffalo, was transferred to the mother house at Emmittsburg, Md., about January 1, 1900. Sister Cynerine, also with the same hospital for seventeen years, was placed in charge of St. Mary's Hospital, at Detroit, Mich. Two Sisters from Emmittsburg took the places made vacant by the transfer of Sisters Florence and Cynerine.

During the Christmas holidays, 1899, the hospital received through the Hon. Wilson S. Bissell $7,000 bequeathed to that institution by the late George Howard Lewis. This early settlement of this bequest was made at the request of Mrs. Lewis, in order that the hospital should receive the money during the holiday season, as it was Mr. Lewis's custom to remember it in the annual distribution of his Christmas charities.

In the beginning of the year 1900, the medical staff was reorganised. Dr. Stephen Y. Howell was chosen president; Dr. Lawrence G. Hanley, vice-president; and Dr. C. M. Daniels, secretary. An executive committee was created consisting of Drs. W. H. Slacer, Byron H. Daggett and Eugene A. Smith.

A training school for orderlies and male nurses was organised at the hospital under the supervision of Dr. B. H. Daggett, who delivered an introductory lecture. Two lectures each week by various members of the staff of physicians and surgeons were established.

In the fall of 1900 the construction of a new Emergency Hospital was commenced on the corner of Eagle and Pine Streets, by John Lannen, contractor. Green and Wicks, were the architects for the new building and the plans called for a three-story brick building with basement. The building was completed on these lines. The basement is used for clinical purposes, the first floor contains the primary wards and the second floor the secondary wards. The surgeons and attendants are housed on the third floor. The Emergency Hospital is the accident branch of the Buffalo Hospital of the Sisters of Charity.

This new hospital was dedicated December 29, 1901, and soon afterward its doors were thrown open for inspection. A little later it was ready for the reception of patients.

Dr. Clayton M. Daniels, of the surgical staff, has recently furnished the *Buffalo Illustrated Express*, (January 12, 1902,) a history of the Emergency Hospital from its beginning from which we learn that the first Emergency Hospital was established in an unpretentious little building at the corner of Pine Street and Booth Alley on October 1, 1883. That institution was in existence exactly six months and during that time it treated 83 cases. The majority of those were railroad cases, as in those days grade crossings improvements and patent couplers were unheard of and the railroads furnished the bulk of the accident business. At the end of the six months it was found that the hospital was not commodious enough for the business and the institution was installed in the building which has just been vacated, at the corner of South Division and Michigan Streets. That was on May 1, 1884. Previous to the opening of the South Division Street institution the surgical work at the Emergency was done by Dr. Daniels and Dr. Byron H. Daggett. After 1884, Dr. Daniels took up the bulk of the surgical work with help from the staff of the Sisters' Hospital.

In the seventeen years it has been occupied as a hospital, the old building on South Division Street sheltered somewhat over 8,000 accident cases. Medical cases have been treated in addition, but the great majority of the cases were casualties, covering almost every range of human affliction. Burns, stabs, poisonings, bullet wounds, asphyxiation, drowning, snake bites, cat bites and dog bites, are some of the causes that contributed to swell the number and it is thought when the total for the entire life of the hospital is known it will show the number of cases to be slightly over 8,000. The total up to May 1st, 1901, was 7,342.

The announcement was made in the summer of 1899 of changes in the staff at St. Mary's Maternity and Infant Asylum, on Edward Street, whereby the institution came under the medical and surgical management of the staff of the Buffalo Sisters of Charity Hospital. Dr. L. G. Hanley was announced as chief obstetrician; the attending obstetricians were Drs. Nelson W.

Wilson, Pierce J. Candee, C. J. Carr and D. V. McClure. Dr. W. H. Slacer was placed at the head of the medical staff. His assistants were Drs. Joseph P. Burke and James Stoddart.

### Buffalo General Hospital.

The Buffalo General Hospital in the spring of 1899 was the recipient of a pleasant surprise, in the nature of a gift of $5,000 from Mrs. Isabella Higgins Moore, of Bethlehem, Pa. This money was invested and the interest therefrom used for the maintenance of a bed known as the "Higgins Memorial Bed." The trustees, in accepting the gift, expressed their appreciation of Mrs. Moore's deep interest in the work of the hospital.

The hospital, about the same time, also received a gift of $1,000 by the will of Milo R. Eames. A resolution was passed thanking the executors and family.

In July, 1899, Dr. Regina Flood Keyes was formally appointed assistant gynecologist on the attending staff. A new position was created, termed assistant to the surgical clinic, and Dr. Edward J. Meyer received this appointment.

In the autumn of 1900 this hospital adopted plans for an addition, the estimated cost of which was $35,000. The additional building is 38 feet wide and 96 feet long. It is an extension of the wing built a few years ago, and corresponds in design and construction to it. The addition fronts on Elm Street. Architect George Cary prepared the plans and Jared H. Tilden built the structure.

### German Hospital.

The German Hospital, located on Jefferson Street, near Genesee, Buffalo, projected in 1895, mention of which is made on page 112, was opened for patients March 11, 1901. It is one of the finest hospitals in the country, whether considered from the viewpoint of construction, equipment, or hygienic arrangement. It has accommodations for about 72 patients, and both wards and private rooms are models of perfection.

The following-named medical and surgical staff was appointed: President, Charles H. W. Auel; vice-president, Marcel Hartwig; secretary, Charles Weil. Internal medicine—consulting physicians—Conrad Diehl, Emil S. Tobie, Thomas Lothrop; attending physicians, H. C. Buswell, William Meisberger, Julius Ullman, Robert Hebenstreit; surgery—consulting surgeon, Roswell Park; attending surgeons, M. Hartwig, Herman Mynter, J. G. Meiden-

THE NEW GERMAN HOSPITAL, JEFFERSON STREET.

bauer, Henry G. Bentz; gynecology—consulting physician, Matthew D. Mann; attending physicians, Charles H. W. Auel, Max Breuer, Herman E. Hayd, Sigmund Goldberg; obstetrics—consulting physician, Charles H. W. Auel; diseases of children, L. Schroeter, Charles Weill, H. C. Rooth; eye and ear—consulting physicians, Lucien Howe, Julius Pohlman; attending physician, Jacob Goldberg; nose and throat, attending physicians, G. F. Cott, W. S. Renner; skin—consulting physician, Ernest Wende; attending physicians, Grover Wende, J. Kraus, Alfred E. Diehl; genito-urinary diseases, Alois Jokl, Julian A. Riester; nervous diseases—consulting and attending physicians, W. C. Krauss, Floyd S. Crego, H. G. Matzinger; pathology, W. G. Bissell, H. R. Gaylord, J. A. Miller.

### Buffalo Fresh Air Mission Hospital.

This hospital was opened at Athol Springs on the lake shore, on July 1, 1894. It was built on plans providing for the most approved system of summer hospital construction, and it is equipped with every modern appliance related to its purposes. Its location is as near ideal as could be, situated on a bluff overhanging Lake Erie, ten miles from Buffalo. It has 30 beds for patients, a commodious administration house with diet kitchen, rooms for the staff, nurses and help, and is reached in 25 minutes from the city.

Dr. Irving M. Snow was the founder of the hospital, at least it was his original suggestion, and it was mainly through his efforts that the proposition to construct such a hospital took root, though others contributed in time and money to work the plans to a conclusion. There are resident physicians, trained nurses, and other assistants on duty at the hospital. Drs. Irving M. Snow and Dewitt H. Sherman have constituted the visiting staff from the first, and one or both make daily visits to the hospital.

It usually opens July 1 each year and remains ready for patients for about ten weeks. It was originally intended for the care and treatment of children suffering from acute summer diarrheas, but its scope has been enlarged to include invalided children in need of country air, convalescents from acute disease, and for the treatment of those afflicted with tubercular bone diseases.

The present staff is as follows: visiting physicians, Irving M. Snow, Dewitt H. Sherman; surgeon, Eugene A. Smith; ophthal-

By courtesy of the Illustrated Buffalo Express,

Copyright by Geo. E. Matthews & Co.

BUFFALO FRESH AIR MISSION HOSPITAL.

mologist, L. Burrows, Jr.; assistant physicians, A. T. Lytle, N. G. Russell.

Number of patients treated in 1901, 90; number of deaths, 7.

The excellent work done by this hospital from its beginning is well known to almost all physicians, and to a large proportion of the inhabitants of Buffalo.

### Pan-American Exposition Hospital.

The officers of the Pan-American Exposition Company began to discuss the formation of a medical bureau for the fair, which should include a well appointed hospital, as early as mid-summer, 1899. The latter project did not take definite shape, however, until after the appointment of Dr. Roswell Park as medical director, which occurred in June, 1900.

EMERGENCY HOSPITAL AT THE PAN-AMERICAN EXPOSITION.

The plans culminated in the construction of a very pretty building near the west end of the mall. Floor area rather than elevation was a prominent feature in the construction, and utility was the prime consideration in the design, though adornment entered into the scheme. In conformity with the general exposition plan the free Spanish renaissance was treated, with a strong leaning toward the old mission interpretation.

With a frontage of 90 feet on the mall, the main wing had a depth of 38 feet with a height of but one story, except in the center, where it assumed the form of a square tower with a rounded top. This tower was two stories high, surmounted with two flagstaffs. One staff supported the exposition flag and the other the Red Cross banner.

A rear wing, one story high, extended back from the center portion a distance of 56 feet, with a width of 32 feet. The main hospital entrance was from the mall, opening directly into a rotunda decorated with tropical plants, pictures and drapery.

The main office was located at the farther left hand corner of the rotunda, under the staircase. It contained telephone, electrical annunciator, messenger call service, and other necessary appurtenances. The first floor front contained in the extreme western wing, two male wards with seven cots each, a bath room, physicians' office, a morgue and a linen chest. The eastern wing contained a ward for women, of a dozen cots, and a bath room. In this wing was an office for the superintendent of nurses, physician's private office, a linen closet and other conveniences.

The upper story was set apart for the use of the resident physician and the necessary attendants, fitted with four pleasant, comfortable bed rooms and a bath room. The rear wing, extending back from the main entrance, contained the operating room, sterilising department and instrument cases. Immediately across the hall was the emergency bath room and patients' waiting room. Still further down the corridor was located the kitchen, pantry and dining room, for the use of patients.

At this hospital the operation was made upon President McKinley, and which was begun within an hour after he was shot at Music Temple. The total number of patients treated at the hospital was 5,567.

The staff was: Dr. Roswell Park, medical director; Dr. Vertner Kenerson, deputy medical director; Dr. Nelson W. Wilson, sanitary inspector; Dr. A. F. Zittel, Dr. Alexander Allan, Dr. G. McK. Hall and Mr. E. C. Mann, resident physicians. Miss Adelia Walters, superintendent of nurses.

### Other Hospitals.

The Buffalo State Hospital which, under the superintendency of Dr. A. W. Hurd, has taken rank among the highest in the country for the treatment of mental diseases, is very much cramped for space,

It has reduced the per capita cost of treatment to the lowest in the state, and has yielded the maximum in results. It is greatly embarrassed by the lack of proper land area, and the propriety of renting a small farm in the vicinity of Buffalo has been discussed, where a limited number of patients could be employed in the care of cows and swine.

A marine hospital station has been proposed at Buffalo. The surgeon-general of the marine hospital service at Washington favors the project and it is hoped and believed that congress will soon pass a bill authorising the construction of a proper hospital. Hon. D. S. Alexander, M.C., has introduced such a bill, appropriating $125,000 for the purpose. Such a building should be centrally located, and it would meet a demand that is constantly growing in this large port.

---

In September, 1899, the Riverside Hospital was removed from 306 Lafayette Avenue, to a new building, corner of Lafayette and Barton Streets, Buffalo. The structure now occupied is well adapted to the needs of the hospital, and the location is desirable. The continued prosperity of the hospital seems assured in its new location. Dr. Lillian C. Randall continues at its head.

---

The private hospital, formerly conducted at 304 Lafayette Avenue, was again opened for the reception of private patients in October, 1899. The management admits only the best class of private patients and aims to give them the best of care and attention. The hospital was newly furnished and made into a first-class private hospital, with all modern improvements and conveniences, and called the Lafayette Hospital. There is no staff of physicians in attendance; each physician sending a case has full control of the patient. Mrs. Lillian C. Lacey, who is in charge, is an experienced nurse and a capable manager.

---

The pavilion for consumptives at the Erie County Hospital was destroyed by fire on the night of March 21, 1900. It was a most unfortunate event, in that it deprived a large number of sufferers of the benefit of that institution. Fortunately, no lives were lost at the fire, but it is probable that the exposure incident to escaping from the burning building shortened the days of some of the patients. The building was of wood and burned so rapidly that some of the inmates barely escaped injury or worse. The lesson—a dear one—means that no building should be constructed for hospital purposes that does not meet all the modern requirements of safety from fire.

A new building was constructed on the old site, at a cost of $50,000, ground having been broken in February, 1901.

---

A new isolation hospital to which should be attached a detention pavilion is among the greatest needs of the health department of Buffalo. Health Commissioner Wende, during his last term of service made urgent demands upon the Common Council for the necessary authority to have such a hospital built, but without definite results.

Buffalo is an important port where hundreds of sailors are arriving and departing daily during the season of navigation, and when one becomes suspected of smallpox or other contagious disease there is no place to assign him pending the development of the case, except to the pest house.

During the summer of 1900, Health Commissioner Wende, was obliged to quarantine two vessels on account of smallpox suspects, much to the loss of the ships, but there was no other way under the existing conditions.

[*To be continued.*]

## Figures and Tables

**Figure f1:**
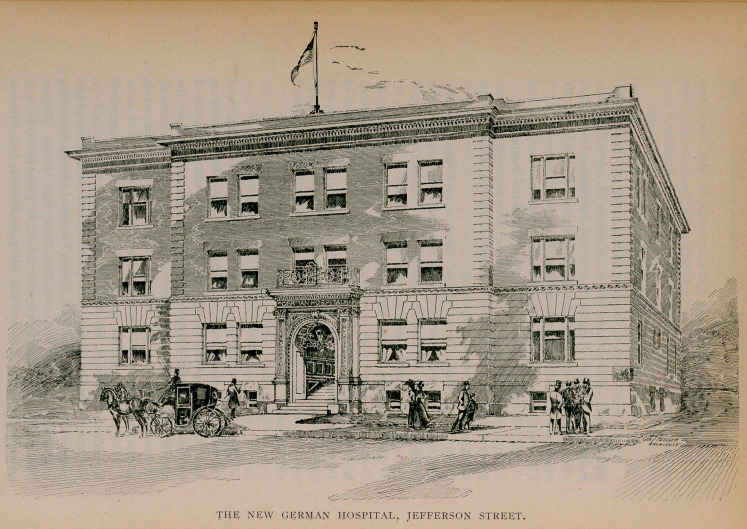


**Figure f2:**
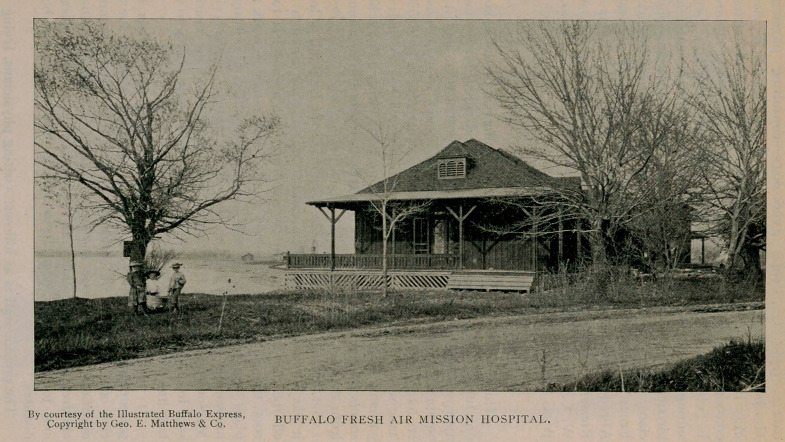


**Figure f3:**